# Copper-Doped Silicate Porous Architectures for Hard Tissue Engineering

**DOI:** 10.3390/jfb17070335

**Published:** 2026-07-09

**Authors:** Cristina Cristea, Maria-Eliza Puscasu, Gabriela-Olimpia Isopencu, Ovidiu-Cristian Oprea, Vasile-Adrian Surdu, Mihaela Bacalum, Roberta Moisa, Sorin-Ion Jinga, Cristina Busuioc

**Affiliations:** 1 Faculty of Medical Engineering, National University of Science and Technology Politehnica Bucharest, RO-060042 Bucharest, Romania; ccristea@stud.fim.upb.ro (C.C.); sorin.jinga@upb.com (S.-I.J.); 2 Faculty of Chemical Engineering and Biotechnologies, National University of Science and Technology Politehnica Bucharest, RO-060042 Bucharest, Romania; maria_eliza.puscasu@stud.fim.upb.ro (M.-E.P.); gabriela.isopencu@upb.ro (G.-O.I.); ovidiu.oprea@upb.ro (O.-C.O.); 3 National Center of Micro and Nanomaterials, National University of Science and Technology Politehnica Bucharest, RO-060042 Bucharest, Romania; 4 Department of Materials Science, Faculty of Materials Science and Engineering, Transilvania University of Brasov, 29 Eroilor Blvd., RO-500036 Brasov, Romania; vasile.surdu@unitbv.ro; 5 Horia Hulubei National Institute of Physics and Nuclear Engineering, RO-077125 Magurele, Romania; mihaela.bacalum@nipne.ro (M.B.); roberta.moisa@nipne.ro (R.M.)

**Keywords:** akermanite, copper doping, 3D printed scaffolds, robocasting, bioactivity, antibacterial activity, bone substitute, tissue engineering

## Abstract

Porous silicate scaffolds represent a promising class of grafting materials for hard tissue engineering due to their superior bioactivity, adjustable degradation rates, and ability to stimulate both osteogenesis and angiogenesis. In this work, scaffolds based on an akermanite-targeted (Ca_2_MgSi_2_O_7_) starting composition, including copper-doped variants, were synthesized using sol–gel and combustion routes, followed by 3D printing to achieve porous architectures with controlled pore size and interconnectivity. The powders were characterized by scanning electron microscopy, energy-dispersive X-ray spectroscopy, Fourier transform infrared spectroscopy, X-ray diffraction, and thermal analysis to evaluate their morphology, composition, and crystalline phases. The scaffolds were further assessed in terms of bioactivity by immersion in simulated body fluid (SBF), antibacterial activity, and in vitro cellular response. The results confirmed that copper doping enhanced antibacterial properties, while maintaining favorable biological behavior. Comparative analysis revealed differences between the two synthesis methods, with sol–gel providing more homogeneous structures and combustion leading to highly porous morphologies. These findings highlight copper-doped silicate scaffolds as promising candidates for bone tissue regeneration, combining architectural integrity with biological functionality.

## 1. Introduction

Tissue engineering and regenerative medicine have gained significant attention over the past decades as strategies to replace or restore damaged tissues through the integration of cells, biomaterials, and bioactive molecules [1,2,3,4,5]. Among the different approaches, scaffolds play a central role as three-dimensional structures that provide physical and biochemical support for cell attachment, proliferation, and differentiation, while gradually degrading to allow tissue replacement. An ideal scaffold must exhibit interconnected porosity, mechanical strength, bioactivity, and controlled biodegradation, making material selection a critical factor in scaffold design [6,7,8,9,10]. One of the fields of regenerative medicine with high demand for synthetic grafts is represented by bone tissue replacement [11,12,13]. Currently, the traditional methods for bone grafting are using the autografts and allografts that have well known limitations [13,14,15]; however, alternatives with high potential could be obtained using the adequate biomaterials and processing methods.

Currently, the most studied bioceramics for bone tissue engineering include calcium phosphates such as hydroxyapatite and tricalcium phosphate, and silicate-based material such as bioactive glasses and calcium and/or magnesium silicates [16,17,18,19,20,21]. While phosphate ceramics are chemically similar to the mineral phase of bone, silicates have demonstrated superior bioactivity, enhanced ability to promote osteogenesis and angiogenesis [22,23,24]. The dissolution of silicate-based biomaterials releases silicon, calcium, and magnesium ions, which stimulate cellular responses and contribute to bone regeneration [25,26,27]. Moreover, silicate ceramics often exhibit improved mechanical strength compared to phosphate analogs, making them attractive candidates for load-bearing applications [22,27]. However, their final mechanical performance strongly depends on the processing route, phase composition, and resulting microstructure.

Within the silicate family, scaffolds based on binary systems such as wollastonite (CaSiO_3_) and dicalcium silicate (Ca_2_SiO_4_) have been investigated for their ability to bond with bone and support cellular activity [28,29]. More recently, ternary silicates that incorporate magnesium, such as diopside (CaMgSi_2_O_6_), merwinite (Ca_3_MgSi_2_O_8_), and akermanite (Ca_2_MgSi_2_O_7_), have received attention due to their dual stimulation of angiogenesis and osteogenesis, controlled degradation, and superior mechanical properties [30]. Among these, akermanite has emerged as a particularly promising candidate, as it combines bioactivity with mechanical stability and displays strong in vitro and in vivo performance in bone regeneration [31]. The formation of a single-phase akermanite structure is inherently challenging due to the competitive reaction pathways occurring during synthesis, which favor the formation of thermodynamically stable binary and ternary silicate phases depending on local chemical environment and processing conditions, as widely reported in the literature. This intrinsic phase competition has also been consistently observed in our long-term experience with solution-based synthesis routes of silicate systems, further confirming the difficulty of achieving phase-pure akermanite under conventional solution-based processing conditions.

Akermanite outstanding performance can be further enhanced by strategic ionic doping to improve its mechanical and biological properties. Incorporation of ions into biomaterials have been demonstrated to enhance bone formation and impact cellular response. There are osteogenic ions (calcium, magnesium, strontium, silicon) that enhance bone formation, angiogenic ions (copper, cobalt) that promote vascularization, antibacterial ions (silver, zinc, copper) that prevent infection, and structure-stabilizing ions (magnesium, strontium, zinc) that improve mechanical strength of the scaffold [32,33,34]. Among the dopants investigated, copper (Cu) has shown significant potential. Copper is an essential trace element in the human body, involved in enzymatic activity, angiogenesis, and bone metabolism [35]. Additionally, copper ions exhibit strong antimicrobial properties by disrupting bacterial membranes, generating reactive oxygen species, and preventing biofilm formation [36]. Incorporating copper into akermanite is therefore expected to provide a dual benefit of enhanced vascularization and antibacterial activity, both crucial for bone regeneration. However, it is well established in the literature that the biological response to copper is dose-dependent, and that local microenvironmental concentrations of released Cu^2+^ ions at the material–tissue interface may transiently reach cytotoxic levels due to burst release effects, even when the overall concentration in the surrounding medium remains lower, thereby affecting cell viability and cellular function through oxidative stress and disruption of cellular homeostasis.

The synthesis of silicate ceramics can be achieved by various methods, among which sol–gel and combustion routes are particularly attractive since they are facilitating the synthesis of powders with small particle sizes. The sol–gel process is a bottom-up wet-chemical method that enables precise control over chemical composition, particle size, and homogeneity, while operating at relatively low processing temperatures; as a result, it allows the fabrication of nanostructured and highly pure powders suitable for bone tissue engineering [37,38]. However, as reported by Alecu et al. [39], obtaining single-phase materials is challenging and requires high control of synthesis and thermal treatment parameters involved in the sol–gel method. In contrast, combustion synthesis is a fast and energy-efficient technique based on a self-sustained exothermic reaction between metal nitrates and organic fuels, producing highly porous powders with fine particle size and large surface area; its simplicity and scalability make it valuable for biomedical ceramics [40,41]. Combustion synthesis of calcium magnesium silicates was studied by Collin et al. [41] and it was reported that it facilitates the preparation of single-phase materials.

The intricate architecture of bone has been demonstrated to play a key role in its mechanical and biological performance. Bone design ensures mechanical strength, shock absorption and appropriate load distribution while being efficiently lightweight. A promising approach in tissue engineering could be to mimic the bone architecture in order to obtain a scaffold with improved mechanical performance [42,43]. However, due to the complex design of the bone, the only manufacturing technique that can allow a high control over the design parameters would be additive manufacturing [44]. Additive manufacturing technologies such as robocasting allow the shaping of scaffolds into porous architectures with precisely controlled pore size, interconnectivity, and geometry [45,46]. This digital approach enables the design of patient-specific implants and overcomes limitations of conventional scaffold preparation methods, ensuring suitability for medical applications that require both mechanical stability and biological functionality [47].

Recent studies have explored copper-doped silicate ceramics, reporting improved bioactivity, angiogenesis, and antibacterial behavior with promising results [35,48]. Copper-doped diopside scaffolds obtained via robocasting were investigated by Peng et al. [46] and it was concluded that the 3D bodies have favorable antibacterial activity increased by the presence of copper. Salahinejad et al. [49] studied the impact of vascularization on scaffolds obtained by incorporating copper-doped akermanite and bredigite in freeze-dried gelatine-based scaffold; the study revealed a significant improvement in angiogenesis generated by the addition of copper-doped akermanite. However, a systematic comparison between akermanite and copper-doped akermanite scaffolds synthesized by both sol–gel and combustion methods and fabricated into 3D porous architectures has not yet been studied to the best of the author’s knowledge.

This manuscript reports the synthesis, characterization, and biological evaluation of scaffolds based on an akermanite-targeted composition and its copper-doped variants, obtained via the sol–gel and combustion methods and subsequently shaped through 3D printing. The novelty of this research lies in: (i) the comparative assessment of two synthesis routes (combustion vs. sol–gel) on phase formation and microstructural evolution; (ii) the investigation of a high copper doping level and its effect on ion incorporation, possible segregation, and release behavior; (iii) the application of extrusion-based 3D printing for shaping the ceramic pastes into architected scaffolds, enabling controlled microporous structures; and (iv) the correlation between morpho-structural features and biological response, including bioactivity and cytotoxicity. These combined aspects provide deeper insight into the optimization of multifunctional silicate-based scaffolds fabrication for hard tissue engineering applications, highlighting the relationship between synthesis route, phase composition, microstructure, printability, ion release behavior, and biological performance.

## 2. Materials and Methods

### 2.1. Materials

The following reagents were used for the synthesis of akermanite (Ca_2_MgSi_2_O_7_) and copper-doped akermanite (Ca_1.65_Cu_0.35_MgSi_2_O_7_): calcium nitrate tetrahydrate (Ca(NO_3_)_2_·4H_2_O, ≥99%, Sigma-Aldrich, St. Louis, MO, USA), magnesium nitrate hexahydrate (Mg(NO_3_)_2_·6H_2_O, ≥99%, Sigma-Aldrich, St. Louis, MO, USA), tetraethyl orthosilicate (TEOS, Si(OC_2_H_5_)_4_, 99%, Sigma-Aldrich, St. Louis, MO, USA), copper(II) nitrate trihydrate (Cu(NO_3_)_2_·3H_2_O, ≥99%, Sigma-Aldrich, St. Louis, MO, USA), glycine (NH_2_CH_2_COOH, ≥99,0%, Sigma-Aldrich, St. Louis, MO, USA), nitric acid (HNO_3_, >65%, Sigma-Aldrich, St. Louis, MO, USA), and ethanol (CH_3_CH_2_OH, ≥99.5%, Sigma-Aldrich, St. Louis, MO, USA). Distilled water was used throughout the experiments. By converting the cationic ratios into mass percentage, a copper content of approximately 7.9 wt% was derived from the nominal stoichiometry of the designed composition. This value is higher than typically reported copper-doping levels in bioactive silicate systems (generally ≤1–5 wt%) and was intentionally selected as an upper-range composition to investigate copper incorporation, possible phase segregation, and its influence on antibacterial and biological performance under high-temperature sintering conditions.

### 2.2. Synthesis Methods

#### 2.2.1. Powder Preparation

Two synthesis routes were employed in this research: solution combustion and sol–gel.

Combustion method: The stoichiometric amounts of calcium, magnesium, and copper nitrates (for doped samples) were dissolved in distilled water at 60 °C, together with glycine (Sigma-Aldrich) as fuel. TEOS was added dropwise under continuous stirring, followed by pH adjustment to 1.5–2.0 using concentrated HNO_3_. The resulting homogeneous solution was subjected to continuous magnetic stirring for 24 h, allowing gradual evaporation of water and formation of a porous gel, consisting of a network of metal and silicon oxides. After gelation, the system was gradually heated with a temperature increase of 30 °C every 30 min until reaching the combustion temperature (~400 °C). The obtained powder was subsequently calcined at 900 °C for 2 h. Finally, the calcined powder was ground in an agate mortar and sieved through a 45 μm mesh to ensure granulometric homogeneity.

Sol–gel method: TEOS was hydrolysed in ethanol (TEOS:EtOH molar ratio 1:3) under ultrasonication for 5 min, followed by pH adjustment to 1.5–2.0 using concentrated HNO_3_. Separately, the stoichiometric amounts of calcium, magnesium, and copper nitrates (for doped samples) were dissolved in distilled water. The TEOS–ethanol solution was added dropwise into the nitrate solution under constant stirring. The obtained mixtures were allowed to form the gel for 24 h, after which they were placed in an oven at 60 °C for 48 h to promote gel maturation and gradual solvent removal. The matured gel was subsequently calcined at 900 °C for 2 h. Finally, the calcined powder was ground with a mortar and sieved through a 45 μm mesh.

#### 2.2.2. Scaffold Fabrication

The silicate-based scaffolds were obtained using the robocasting technique. The 3D printing process implies mixing powders with an appropriate additive to obtain a paste with adequate rheological performance for the extrusion process. The agent mixed with the silicate-based powders was represented by an aqueous solution of hydroxypropyl methylcellulose (HPMC, Sigma-Aldrich) with a 10 wt% concentration. The impact on the powder-to-additive ratio, determined by the synthesis method, as well as the presence of dopant in the paste formulation, was also assessed.

**Figure 1:**
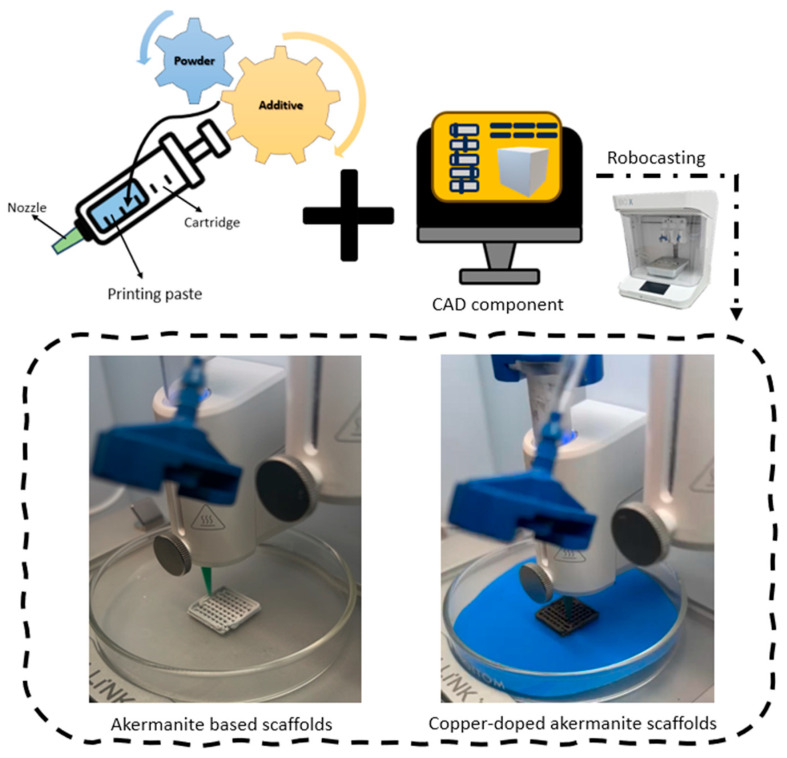
Schematic representation of the robocasting process used for scaffold fabrication, together with representative photographs of the printing process for the undoped and copper-doped ceramic pastes.

The resulting paste was extruded through a 0.8 mm nozzle to produce scaffolds with dimensions of 20 mm × 20 mm × 3 mm. A regular 0°/90° grid architecture was selected to generate an interconnected porous network that could promote nutrient diffusion, cell migration, and tissue ingrowth while maintaining adequate structural stability. The infill of 33% was considered to ensure a porosity appropriate for tissue regeneration processes. The printing speed was set to 15 mm/s, with printing pressures between 100 and 200 kPa. The printing process of the silicate-based scaffolds is displayed in Figure 1.

The obtained green bodies were subsequently sintered at 1200 °C for 3 h. The samples were heated at 2 °C/min up to 400 °C and held for 60 min to ensure controlled removal of HPMC, preventing pore formation and cracking. The temperature was then increased at 5 °C/min to 1200 °C, where it was maintained to achieve densification, grain growth, and structural consolidation. This sintering temperature was selected to remain below the melting temperature of akermanite (~1350 °C), while accounting for the potential fluxing effect of copper and the increased reactivity of the powders obtained via wet-chemistry synthesis routes. A limitation of the present study is that shrinkage occurring during drying and/or sintering was not quantitatively evaluated; therefore, its effect on the final scaffold dimensions will be investigated in future studies. The obtained samples were coded according to the data in Table 1.

### 2.3. Characterization Methods

The morphology and elemental composition of powders and 3D scaffolds were evaluated by scanning electron microscopy (SEM) with a FEI Quanta Inspect F50 (FEI Company, Hillsboro, OR, USA) operated at 30 kV with a 10 mm working distance. Samples were sputter-coated with a thin gold layer (DC sputtering, 60 s) to ensure conductivity. SEM was coupled with energy-dispersive X-ray spectroscopy (EDX) to determine the elemental distribution in the ceramic matrix.

Fourier Transform Infrared Spectroscopy (FTIR) spectra were recorded with a Thermo Scientific Nicolet iS50 spectrometer (Thermo Fisher Scientific, Waltham, MA, USA), using the attenuated total reflectance (ATR) mode. Spectra were collected in the range 400–4000 cm^−1^, at 4 cm^−1^ resolution, with 32 scans per sample. This method enabled identification of functional groups, silica network bonds, and potential organic residues.

Phase composition and crystallinity were investigated using X-ray diffraction (XRD) technique, carried out at room temperature with Shimadzu XRD 6000 (Shimadzu Corporation, Kyoto, Japan) with Cu K*α* radiation (*λ* = 1.5406 Å) filtered by Ni. Data were collected in the 2*θ* range 20–80° with a step size of 0.02° and scan speed of 2°/min. Rietveld refinement conducted in HighScore Plus v3.0e software (Malvern Panalytical, Malvern, UK) was applied to quantify crystalline phases and estimate crystallite size; background fitting was performed using a polynomial function, whereas FWHM approximation was obtained using a pseudo-Voigt profile and the Caglioti function, respectively. The sample crystallinity was evaluated from the ratio of the integrated diffraction peak intensity to the total measured intensity.

The thermal behavior of the powders was evaluated by thermal analysis (TGA/DSC) (Netzsch STA 449 F3 Jupiter, Selb, Germany). Measurements were conducted in air atmosphere, from room temperature to 900 °C, with a heating rate of 10 °C/min. This analysis provided data on weight loss, decomposition processes, and phase transitions.

Bioactivity was evaluated by immersing samples in simulated body fluid (SBF), prepared according to Kokubo’s protocol [50], at 37 °C for up to 28 days. Surface modifications and apatite layer formation were monitored by SEM, confirming the osteoinductive potential of the materials.

Antimicrobial properties were tested against *Escherichia coli* (DH5K, Gram-negative) and *Bacillus subtilis* (ATCC 6633, Gram-positive). Bacteria were cultured on Luria–Bertani (LB) medium (Roth, Germany). The plates were inoculated with bacterial suspension (EC–*DO* = 0.3875, BS–*DO* = 0.2375) by the inoculum depletion method. The plates were left for about 1 h in the oven with controlled humidity, so that the bacterial suspension is uniform impregnated in the medium and there is no excess liquid in the plate. Samples, as small fragments (a few millimeters in size) and with similar mass, were sterilized under UV light (256 nm, 30 min, UV lamp, ROTH, Germany) and placed on the surface of the medium in the Petri dishes. The probes were incubated for 24 h at 37 °C. The antibacterial activity of the scaffolds was evaluated by measuring the inhibition zone (*IZ*, mm) represented by the clear area immediately adjacent to the scaffold where bacterial growth was completely suppressed and the halo zone (*HZ*, mm) that corresponds to the surrounding regions of reduced bacterial growth. Zone widths were calculated from the measured diameters after subtracting the scaffold edge length.

Biocompatibility was tested using human fetal osteoblast cells (hFOB 1.19, ATCC, Manassas, VA, USA). Cells were cultured in a 1:1 mixture of Ham’s F12 and DMEM media (Gibco, Waltham, MA, USA), supplemented with 10% fetal bovine serum (FBS, Gibco, USA) and 1% penicillin–streptomycin. Viability and adhesion were assessed after 48 h of incubation at 37 °C in a humidified atmosphere with 5% CO_2_, evaluating cell proliferation on the scaffold surfaces. For viability assay, following the desired time of growth, the medium was replaced with a 1 mg/mL MTT solution (Serva, Heidelberg, Germany) and further incubated for 3–4 h at 37 °C. Then, the medium was removed, and the formazan crystals formed were dissolved using DMSO. After 30 min the solution absorbance was measured at 570 nm using a Mithras LB 940 plate reader (Berthold Technologies, Bad Wildbad, Germany). Finally, the cell viability was calculated relative to cells grown on a glass slide. All experiments were repeated at least three times. For the morphological changes, the cytoskeleton and nucleus of the cells were analyzed. Staining the actin filaments was done using Phalloidin-FITC (Sigma-Aldrich, Saint Louis, MO, USA) and for the nucleus Hoechst 33342 (Thermo Fisher Scientific, Waltham, MA, USA) was used. The images were taken using an Olympus BX-51 epifluorescence microscope (Olympus, Dusseldorf, Germany), equipped with a 40× objective and DAPI/Hoechst and GFP/FITC filters. In short, the cells were washed with phosphate-buffered saline (PBS) three times, fixed with 4% formaldehyde, rewashed three times with PBS, permeabilised with 0.1% Triton X-100 in PBS, and washed again with PBS three times. Finally, the fluorescent dye was added to the cells and left in the dark, at room temperature, for 1.5 h. In the last step, the cells were washed with PBS three times and fixed with FluorSaveTM (Merck, Darmstadt, Germany). The images were further pseudo-colored using the ImageJ software (version 1.53a, Madison, WI, USA).

In terms of statistical analysis, the data was evaluated using Prism software version 9.0.0. The data were analyzed for a paired comparison of individual data means by one-way ANOVA analysis. A *p*-value < 0.05 was considered statistically significant. All the experiments were conducted three times and data were represented in mean ± SD.

## 3. Results and Discussion

### 3.1. Powder Characterization

**Figure 2:**
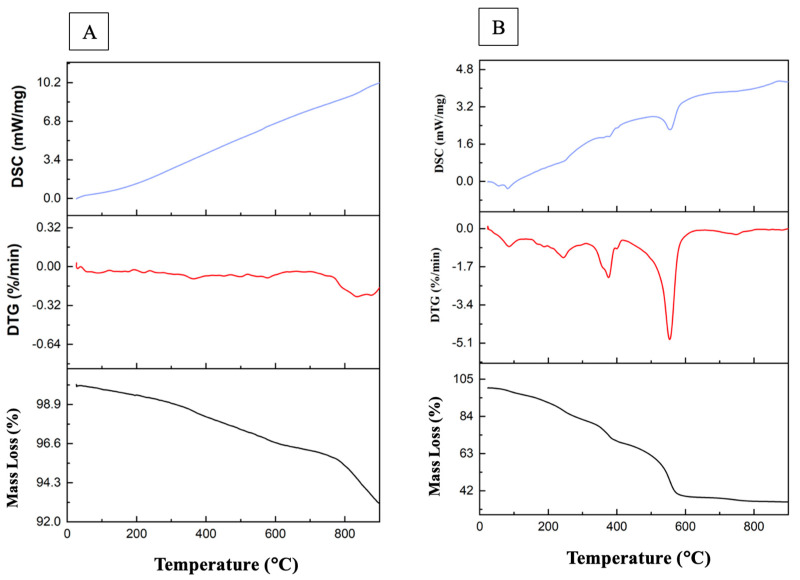
Thermal analysis (mass loss, DTG—differential thermogravimetry and DSC—differential scanning calorimetry) of the uncalcined powders obtained by: (**A**) combustion method and (**B**) sol–gel method.

The thermal behavior of the powders is shown in Figure 2. Both analyses included three curves: mass loss (TGA), differential thermogravimetry (DTG), and differential scanning calorimetry (DSC). For the combustion-derived sample (Figure 2A), the total weight loss was relatively small (~7%, from 100 to 93% in the range 26–900 °C), with a slight step above 700 °C. The flat DTG curve and the smooth, ascending DSC signal indicated the absence of sharp decomposition events, confirming the high thermal stability and partial crystallization of the powders during the combustion reaction. This limited mass loss can be attributed to residual species originating from the combustion synthesis route, including residual solvents, unreacted or partially decomposed combustion fuel (glycine), nitrate-derived residues, and organic-related species associated with TEOS. Nevertheless, as reported by Collin et al. [41], post-synthesis thermal treatment is still required to obtain a well-crystallized akermanite phase.

In contrast, the sol–gel powders exhibited a major weight loss of ~64% (from 100 to 36%), due to the elimination of solvents, organics, and nitrates (Figure 2B). The TGA/DTG curves revealed multiple stages: removal of adsorbed water below 130 °C, decomposition of organic precursors in the range 130–310 °C, and the main mass loss between 400 and 600 °C, corresponding to complete burnout of organics and nitrates. The pronounced DTG minimum at ~550 °C and the strong endothermic DSC peak at ~560 °C confirmed structural reorganization and crystallization of silicate phases. Above 600 °C, the curves stabilized, indicating attainment of a stable crystalline phase. These observations are consistent with reports that sol–gel-derived material initially forms as an amorphous or weakly crystalline gel that requires significant post-synthesis thermal treatment to achieve complete crystallization, as previously shown by the work of Mihailova et al. [51] and Alecu et al. [39].

**Figure 3:**
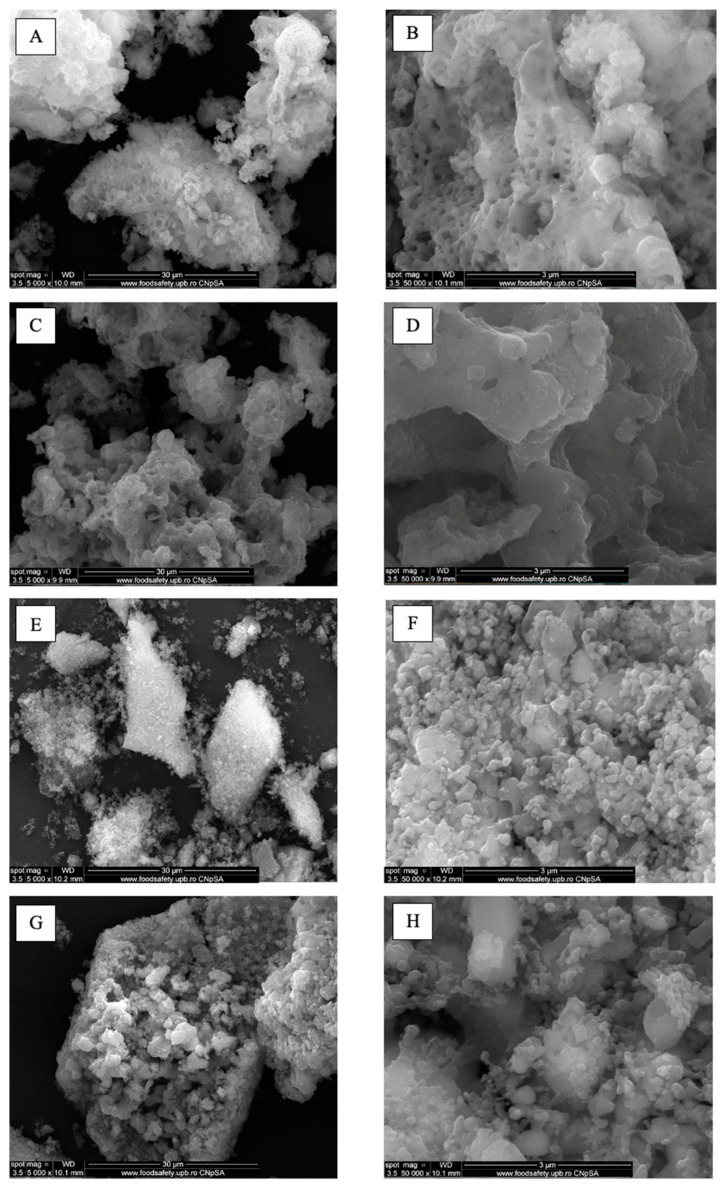
SEM images at 5000× and 50,000× magnifications of the powders calcined at 900 °C: (**A**,**B**) Ak-C-900, (**C**,**D**) Ak-Cu-C-900, (**E**,**F**) Ak-Sg-900, and (**G**,**H**) Ak-Cu-Sg-900.

Figure 3 shows the SEM images captured on the Ak-C-900, Ak-Cu-C-900, Ak-Sg-900 and Ak-Cu-Sg-900 powders. For Ak-C-900 powder, the SEM images at different magnifications evidence the presence of agglomerated particles with a highly porous, foam-like morphology, with irregularly shaped grains. Figure 3B reveals that the surface of the combustion-derived particles appears irregular and highly porous, with randomly distributed non-homogeneous pores, suggesting a large specific surface area. Similar morphological trends for powders obtained by combustion can be corelated with the results obtained by Collin et al. [41], who reported the sponge-like morphology with non-homogeneous pores for the akermanite powder synthesized by combustion method, while Kothandam et al. [52] concluded that the fuel used in the combustion process impacts the morphology of the powder, the non-homogeneous behavior being attributed to the use of glycine. In contrast, the Ak-Sg-900 powder displays more regular features, with homogeneous particle distribution of flat and prismatic particles with decreased particles size, assembled into clusters. The morphology of the Ak-Sg-900 sample in Figure 3D suggests that the sol–gel powder also displays surface roughness, but with more defined particle outlines, indicative of a more controlled growth and densification and consistent with the work of Alecu et al. [39] on sol–gel-derived calcium magnesium silicates thermally treated at various temperature ranging from 600 to 1300 °C. The morphological differentiation between the Ak-C-900 and Ak-Sg-900 is likely given by the nucleation and growth dynamics during gel formation and thermal processing.

For the Ak-Cu-C-900 and Ak-Cu-Sg-900 samples, the SEM images show that by both combustion and sol–gel synthesis ensure the persistence of significant particle agglomerations; however, the copper-doped powders tend towards reduced pore size and a moderately denser microstructure compared to their undoped counterparts. The differences in morphology for the copper-doped powders confirm that the dopant incorporation influences particle coalescence. Similar behavior in copper-doped powders was reported by Htun et al. [48], who demonstrated that copper doping influences grain growth and densification in akermanite ceramics.

**Figure 4:**
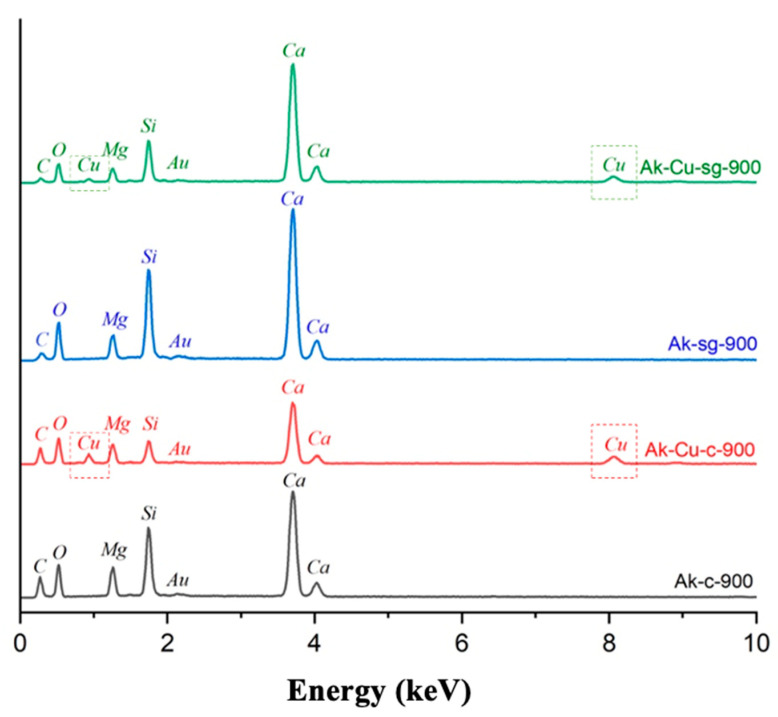
EDX spectra of the powders calcined at 900 °C (Ak-C-900, Ak-Cu-C-900, Ak-Sg-900 and Ak-Cu-Sg-900).

EDX spectra of the Ak-C-900, Ak-Cu-C-900, Ak-Sg-900 and Ak-Cu-Sg-900 powders synthesized by both routes show clear evidence of calcium (Ca), magnesium (Mg), silicon (Si), and oxygen (O) peaks, consistent with the expected elemental constituents of a ternary silicate. The distribution of elements appears uniform over the entire analyzed surface, which equals to a good homogeneity of the material for both synthesis methods. In copper-doped powders, an additional copper (Cu) peak is noticed in the EDX spectra, a fact that confirms the incorporation of copper during the synthesis process in both Ak-Cu-C-900 and Ak-Cu-Sg-900 samples (Figure 4).

**Figure 5:**
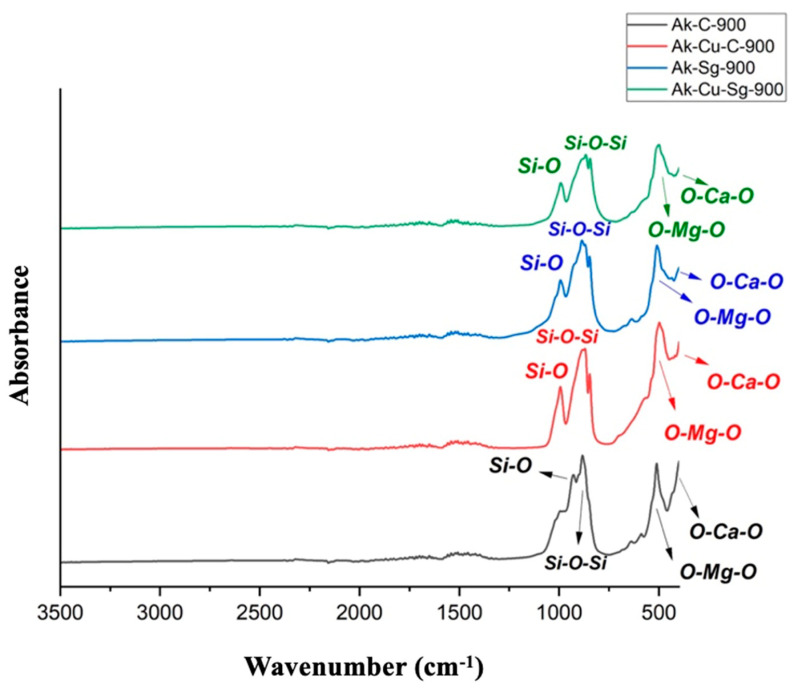
FTIR spectra of the powders calcined at 900 °C: (Ak-C-900, Ak-Cu-C-900, Ak-Sg-900 and Ak-Cu-Sg-900).

FTIR spectra of the synthesized powders are presented in Figure 5. Both sol–gel and combustion samples showed characteristic Si–O–Si stretching vibrations at ~900–1100 cm^−1^ and Si–O bending modes at ~450 cm^−1^, confirming the formation of silicate networks, as also reported by Alecu et al. [39]. Bands attributed to Ca–O and Mg–O bonds were observed in the 500–600 cm^−1^ region. For the doped samples, a slight shift in band positions was detected, suggesting structural modification upon copper incorporation, results also found in the work of Yousef El Adawy et al. [35].

**Figure 6:**
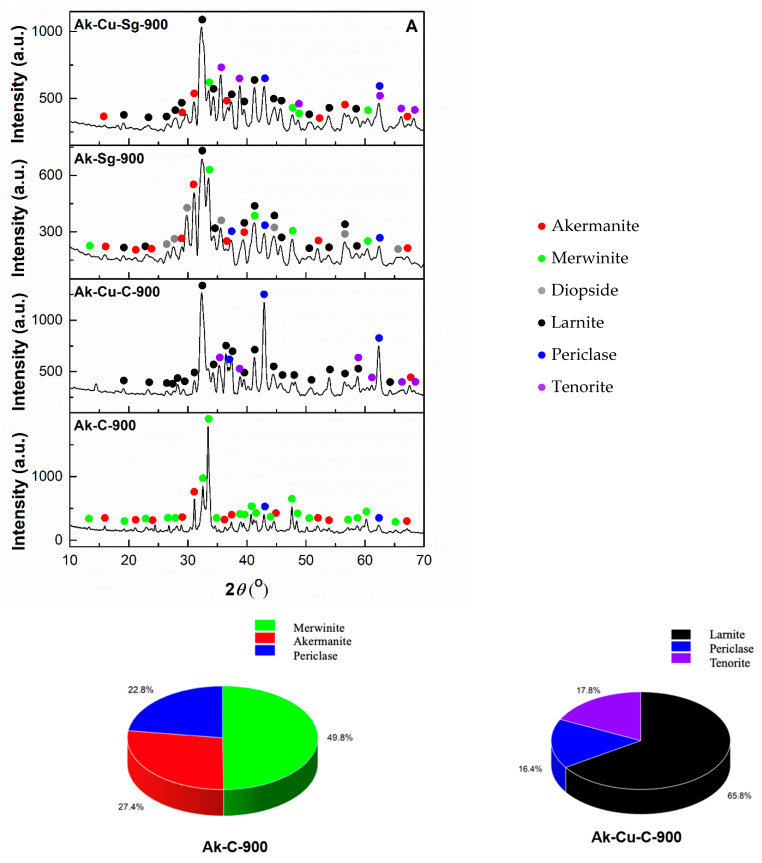

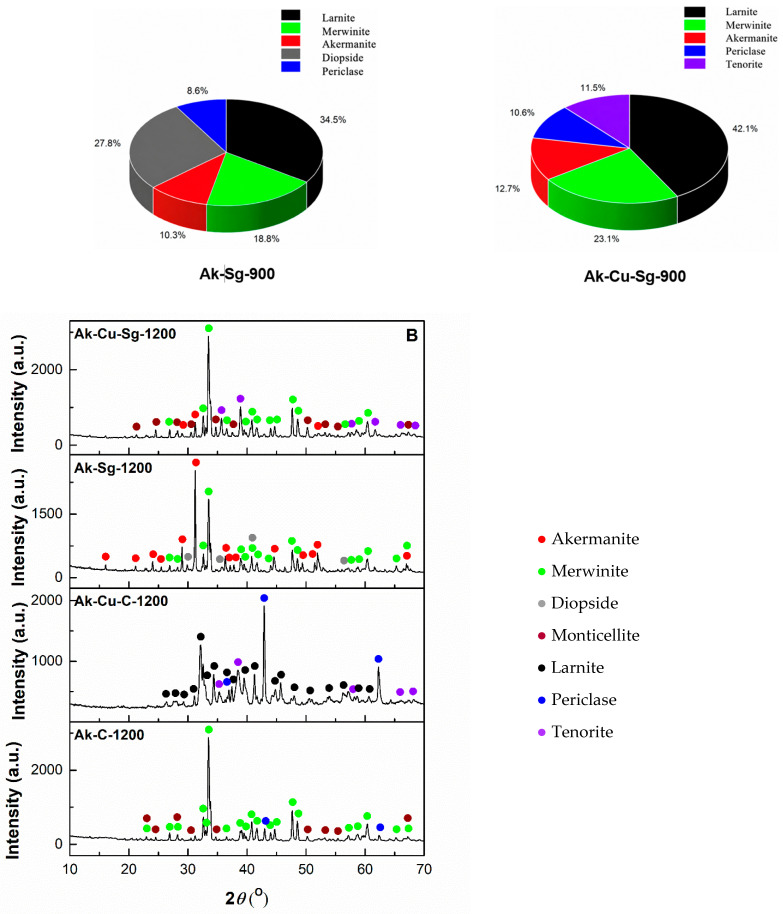

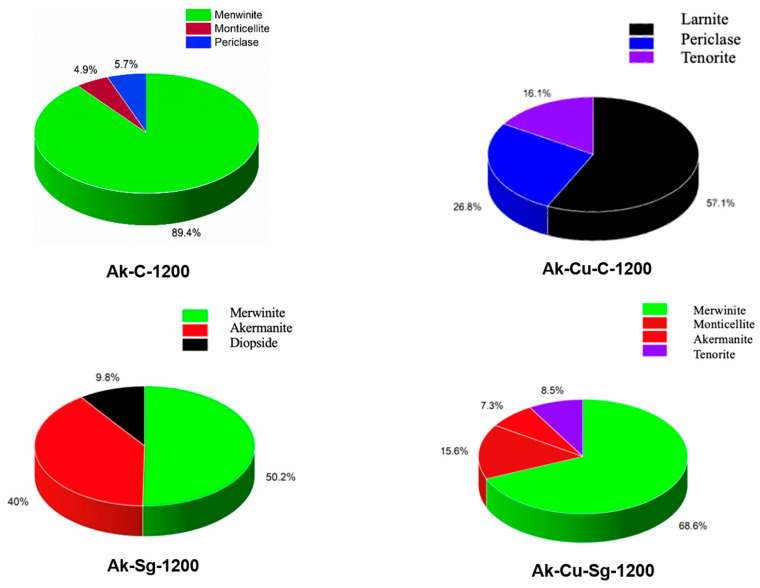
XRD pattern of: (**A**) the powders calcined at 900 °C (Ak-C-900, Ak-Cu-C-900, Ak-Sg-900 and Ak-Cu-Sg-900) and (**B**) scaffolds sintered at 1200 °C (Ak-C-1200, Ak-Cu-C-1200, Ak-Sg-1200 and Ak-Cu-Sg-1200), together with the corresponding phase composition determined by Rietveld refinement and presented as pie charts.

XRD analyses recorded for both powders and scaffolds (Figure 6A) reveal a significant structural impact given by the synthesis method and the dopant presence for the Ak-C-900, Ak-Sg-900, Ak-Cu-C-900 and Ak-Cu-Sg-900 powders. The results of the Rietveld refinement performed on the corresponding XRD patterns are listed in Table 2, offering a more detailed insight in the phase composition. For the undoped combustion-derived sample thermally treated at 900 °C, multiple crystalline phases were detected, with merwinite (Ca_3_MgSi_2_O_8_, monoclinic crystal system, ICDD 96-900-0286) being dominant, alongside akermanite (Ca_2_MgSi_2_O_7_, tetragonal crystal system, ICDD 96-900-6449) and residual periclase (MgO, cubic crystal system, ICDD 96-900-0502), revealing incomplete reaction of precursors at 900 °C. These findings are in contradiction with the data reported by Collin et al. [41], where it is indicated that above 800 °C the tendency is towards formation of a single-phase akermanite-based material, with complete removal of impurities. This discrepancy may be an indicator that the time required for mixing the precursors and the heating rate still need to be optimized [53]. In contrast, the Ak-Sg-900 powder exhibits a more favorable phase composition at 900 °C, with merwinite, akermanite, diopside (CaMgSi_2_O_6_, monoclinic crystal system, ICDD 96-900-0332) and larnite (Ca_2_SiO_4_, monoclinic crystal system, ICDD 96-901-2793) crystalline phases coexisting. The obtained data for the Ak-Sg-900 sample can be correlated with the research of Alecu et al. [39] and Nicoara et al. [54], where it is reported that the sol–gel route leads to the formation of mixtures of several crystalline phases even at high temperature, with the thermal treatment playing a key role in controlling the phase composition. For the copper-doped powders, the phase assemblage shifted significantly.

The Ak-Cu-C-900 powder contains mainly larnite, periclase, and tenorite (CuO, monoclinic crystal system, ICDD 96-901-5842), with limited akermanite formation, while the Ak-Cu-Sg-90 sample shows a more balanced distribution of phases at 900 °C. The presence of tenorite confirms copper incorporation, although not fully into the akermanite lattice. The obtained result for the copper-doped materials cannot be correlated with the data published by Htun et al. [48], who concluded the fact that copper provides structural stability and no additional phase formation was noticed, once again this being an indicator of the thermal treatment importance. These results demonstrate that the sol–gel method is more effective for obtaining akermanite, while copper doping modifies the equilibrium by favoring secondary phase formation.

Overall, the multiphase composition is expected to significantly influence the final mechanical and biological performance through variations in surface energy, phase solubility, ion release behavior, and microstructural heterogeneity, which collectively govern dissolution kinetics, interfacial stability, mechanical reliability, and bioactivity, while simultaneously reducing stiffness and load-bearing capacity due to increased phase complexity. The identified phases provide complementary functions within the system: calcium magnesium silicates (monticellite, diopside, akermanite and merwinite) contribute to bioactivity and structural stability, larnite promotes rapid ion release and apatite formation, while periclase and tenorite act as functional phases influencing local alkalinity and antibacterial performance, respectively, resulting in a synergistic multiphase system with tunable properties.

### 3.2. Scaffold Characterization

Considering the above data, the green scaffolds obtained in this research were subsequently sintered at 1200 °C, with the corresponding XRD analyses being displayed in Figure 6B. The XRD patterns and the results of the Rietveld refinement (Table 2) demonstrate that for the Ak-C-1200 scaffold, merwinite becomes the main phase (~89%), while akermanite disappears, being replaced by monticellite (CaMgSiO_4_, orthorhombic crystal system, ICDD 96-900-1075), while the amount of periclase is strongly reduced, confirming advanced crystallization, but not achievement of the target phase. For the Ak-Sg-1200 sample, akermanite reaches ~40% of the phase composition, with well-defined diffraction peaks, suggesting that the sol–gel route promotes the formation of the desired crystalline phase more effectively. The Ak-Cu-C-1200 scaffold contains mainly larnite, periclase, and tenorite, with an increase in larnite concentration compared to the sample thermally treated at 900 °C, while the Ak-Cu-Sg-1200 sample shows a more balanced distribution of phases and higher crystallinity at 1200 °C.

**Figure 7:**
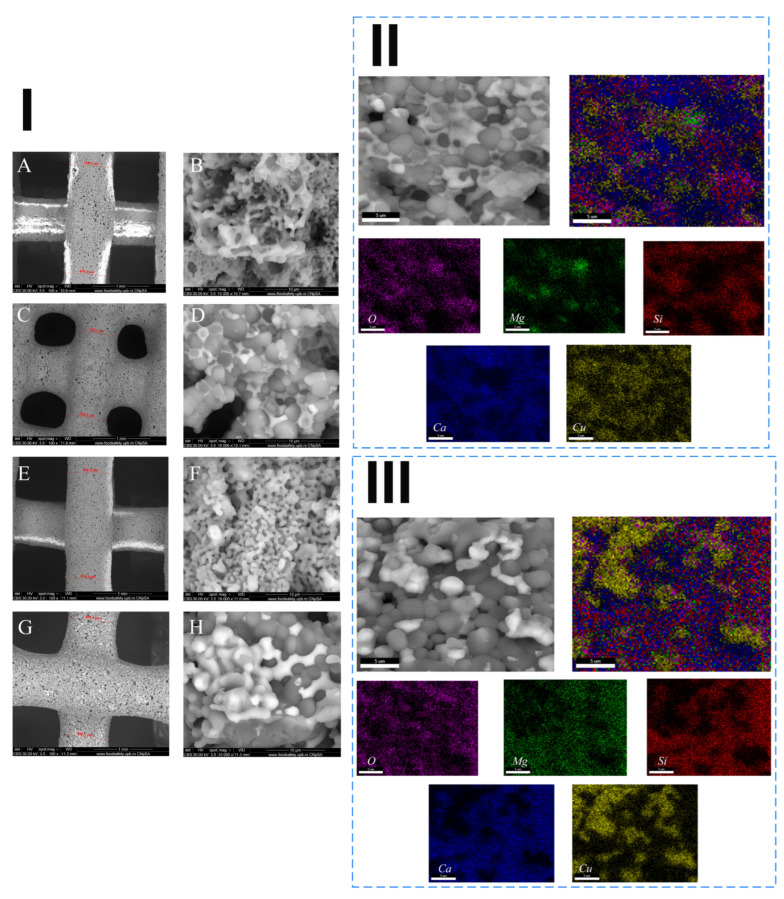
(**I**) SEM images at 1000× and 10,000× magnifications of the scaffolds sintered at 1200 °C: (**A**,**B**) Ak-C-1200, (**C**,**D**) Ak-C-Cu-1200, (**E**,**F**) Ak-Sg-1200 and (**G**,**H**) Ak-Cu-Sg-1200, (**II**) SEM image and corresponding elemental distribution maps of the Ak-C-Cu-1200 scaffold, and (**III**) SEM image and corresponding elemental distribution maps of the Ak-Sg-C-1200 scaffold. Elemental distribution maps are shown both as overlaid and individual images.

Representative SEM images of the 3D printed scaffolds are shown in Figure 7I. Both combustion and sol–gel-derived scaffolds displayed a porous microstructure with uniformly distributed pores, generated by the burnout of the organic binder in the sintering process. However, the sol–gel scaffolds exhibited smaller, more uniformly distributed pores, while the combustion-derived ones had a rougher surface and larger, irregularly shaped pores due to residual combustion gases. Copper doping led to slightly denser microstructures, which may improve mechanical integrity. The Ak-Cu-Sg-1200 scaffolds demonstrated slightly higher densification and finer grain size, confirming a more efficient sintering process and better structural uniformity. The strand thickness is close to 0.8 mm for all the printed samples thermally treated at 1200 °C, which confirms that the paste composition and printing parameters were appropriately optimized for the extrusion process. A variation in the paste formulation was noted, depending on the synthesis route and dopant presence. The Ak-C-900 powder involved a 1.00:1.00 ratio of powder to additive, while the Ak-Sg-900 sample required less additive for the same amount of powder, with a ratio of 1.25:1.00. In general, the copper-doped powders required a higher amount of additive than the undoped ones, with a 25% increase in the additive required for both compositions (Ak-Cu-C-900 and Ak-Cu-Sg-900) to obtain pastes with adequate behavior for the extrusion purpose. The backscattered electrons (BSE) used to obtain the SEM images show contrast variations that are in consonance with the phase mixtures identified through the XRD analysis. The corresponding images highlight the complex multiphase assemblage present in the final scaffolds, which was determined by the XRD investigation as a mixture of merwinite, akermanite, diopside, monticellite, larnite and periclase, in different amounts, as centralized in Table 2. Simultaneously, for the copper-doped materials, the brighter areas in the SEM images obtained with BSE can be attributed to the tenorite phase [55].

The elemental distribution maps obtained by EDX for the sintered copper-doped scaffolds are presented in Figure 7II,III. The elemental mapping of the combustion-derived scaffold shows the presence of the major elements calcium (Ca), magnesium (Mg), silicon (Si), oxygen (O), and copper (Cu). Calcium appears as the predominant and uniformly distributed element, while copper exhibits a generally homogeneous distribution with a few localized areas of higher concentration, suggesting partial segregation. In contrast, the sol–gel-derived scaffold shows a slightly higher copper aggregation accompanied by a more uniform distribution of magnesium. The thermal analysis, XRD and EDX investigations collectively indicate the partial incorporation of copper in the lattice of different silicates through substitutional mechanism by both combustion and sol–gel synthesis methods. The presented results also highlight the clear impact of the synthesis route on the phase evolution, crystallinity variation and microstructure heterogeneity.

**Figure 8:**
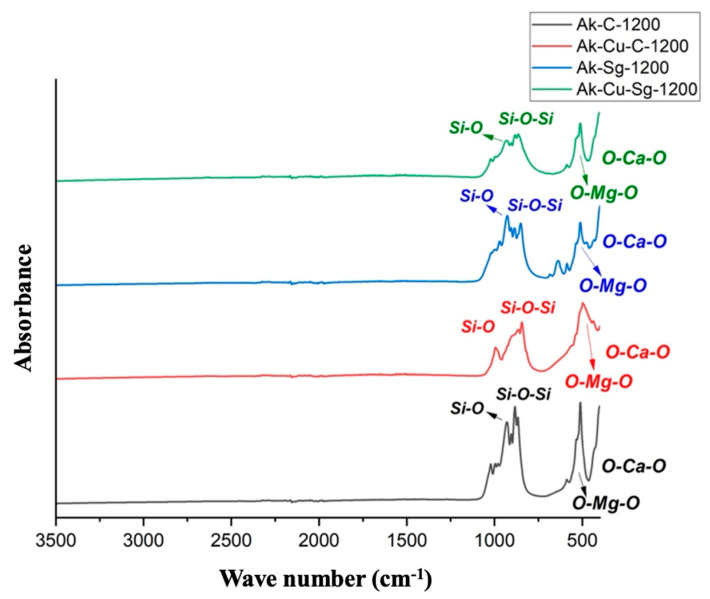
FTIR spectra of the powders sintered at 1200 °C (Ak-C-1200, Ak-Cu-C-1200, Ak-Sg-1200 and Ak-Cu-Sg-1200).

The FTIR spectrum of the sintered samples (Figure 8) revealed the characteristic absorption bands corresponding to calcium and magnesium silicate structures. The characteristic Si–O–Si stretching vibrations at ~900–1100 cm^−1^ and Si–O bending modes at ~450 cm^−1^, together with the bands attributed to Ca–O and Mg–O bonds observed in the 500–600 cm^−1^ region are still present in the spectra. Upon increasing the temperature of the thermal treatment from 900 to 1200 °C, the bands associated with the Si–O vibrations become narrower and sharper for all samples, a fact that reflects an increase in crystallinity, influenced by the progressive transformations occurred in the silicate network, as well as by the transition from binary to ternary silicates, aspects reported in the scientific literature as well [56].

**Figure 9:**
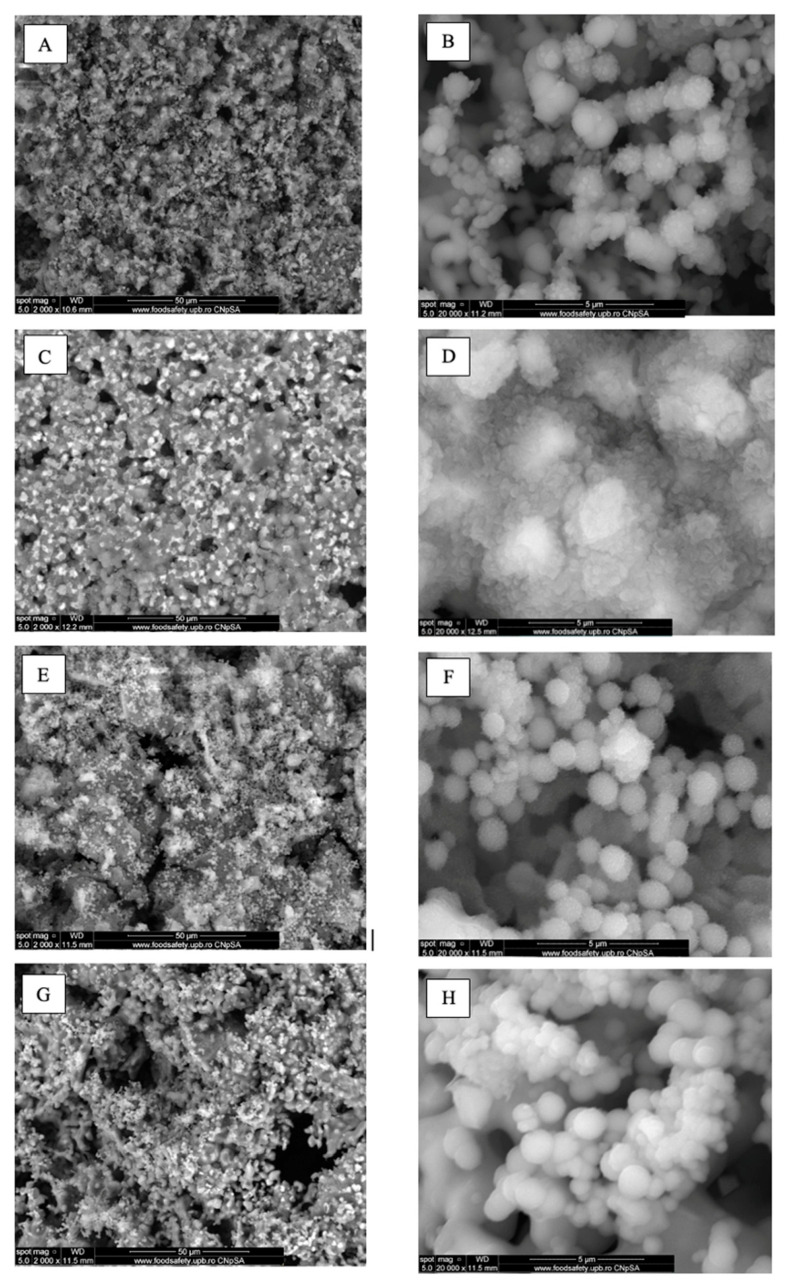
SEM images at 2000× and 20,000× magnifications of the scaffolds sintered at 1200 °C, immersed for 4 weeks in SBF: (**A**,**B**) Ak-C-1200, (**C**,**D**) Ak-Cu-C-1200, (**E**,**F**) Ak-Sg-1200, and (**G**,**H**) Ak-Cu-Sg-1200.

After 4 weeks of immersion in simulated body fluid (SBF), the surfaces of all scaffolds were almost completely covered by a layer of spherical entities with a morphology resembling cauliflower and diameters between 0.5 and 1.0 μm, indicating the formation of a bone-like apatite layer on the surface of the Ak-C-1200, Ak-Cu-C-1200, Ak-Sg-1200 and Ak-Cu-Sg-1200 scaffolds (Figure 9). This mineral layer confirms the materials’ bioactivity and their ability to interact with the physiological fluids and induce bone-like apatite formation. The obtained data correlates with the work of Tavangarian et al. [57], Najafinezhad et al. [58] and Yousef El Adawy et al. [35], who previously reported that akermanite has an increased bioactivity, with high potential in bone regeneration. It was also noticed that the combustion-derived scaffolds exhibited a denser and more uniform apatite coverage compared to the sol–gel-derived ones. In particular, the Ak-Cu-C-1200 sample showed the most distinctive surface morphology, being the only sample exhibiting a continuous, nanostructured apatite layer, whereas the other samples presented discrete, globular apatite deposits dispersed on the surface. This behavior suggests a faster ion release and local supersaturation, which can promote apatite nucleation and early-stage mineralization. Although the apatite layer appears more compact in the case of this sample, the overall amount of mineral deposition may be lower compared to the samples showing globular and more densely distributed deposits, indicating that surface coverage continuity does not necessarily correlate with the total quantity of formed apatite. Notably, this enhanced ion release behavior, particularly for the combustion-derived copper-doped samples, is also consistent with the cytotoxicity results discussed later in the manuscript, where higher ion availability correlates with reduced cell viability.

**Figure 10:**
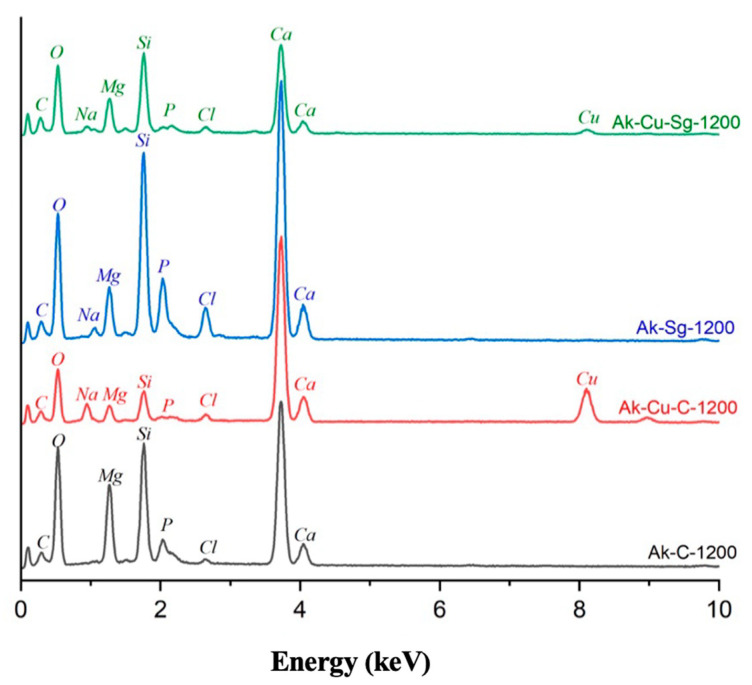
EDX spectra of the scaffolds sintered at 1200 °C, immersed for 4 weeks in SBF (Ak-C-1200, Ak-Cu-C-1200, Ak-Sg-1200 and Ak-Cu-Sg-1200).

EDX analysis of the surfaces after immersion (Figure 10) revealed significant calcium (Ca) and phosphorus (P) peaks, confirming the formation of calcium phosphate phases. The higher phosphorus intensity observed in the sol–gel-processed scaffolds suggests a more pronounced mineralization; however, this observation should be interpreted with caution considering the limitations of EDX analysis, which does not allow a fully reliable quantitative evaluation based on peak intensities alone, particularly in the presence of compositional heterogeneity and element segregation effects. Therefore, the observed differences in intensity can be considered semi-qualitative indicators rather than precise measures. This behavior may be associated with the smaller grain size, which facilitates a higher nucleation density, or with differences in ion release related to phase composition. Simultaneously, the undoped samples showed slightly higher bioactivity compared to the copper-doped ones. The decrease in mineralization kinetics can also be correlated with the available research data, where it is reported that copper doping reduces apatite formation, as copper ions may partially inhibit apatite nucleation due to copper ions competing with calcium ions for calcium phosphate precipitation, as described by Vergnaud et al. [59]. However, research data also indicates that although mineralization is slowed down, the copper-containing calcium magnesium silicate systems still maintain an adequate bioactivity [59,60].

**Figure 11:**
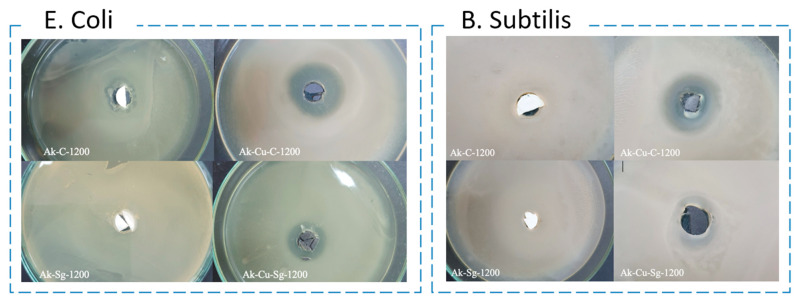
Digital images illustrating the antibacterial effect of the scaffolds sintered at 1200 °C (Ak-C-1200, Ak-Cu-C-1200, Ak-Sg-1200 and Ak-Cu-Sg-1200) against *E. coli*. and *B. subtilis* after 24 h of incubation.

The antibacterial activity of the scaffolds was evaluated by measuring the inhibition zone, the clear area around the sample where bacterial growth was completely inhibited, and the halo zone, corresponding to regions of reduced bacterial growth. As shown in Figure 11 and Table 3, the copper-doped scaffolds exhibited significantly higher antimicrobial activity compared to undoped ones, against both *E. coli* and *B. subtilis*. The effect was more pronounced for *E. coli*, likely due to the negatively charged outer membrane of Gram-negative bacteria, which enhances their interaction with released copper ions. Copper ions act through multiple mechanisms, including disruption of bacterial membranes, interference with metabolic pathways, and generation of reactive oxygen species (ROS) that damage cellular components and DNA, leading to cell death [61]. The data is consistent with the paper published by Sheng et al. [62], where it is indicated the fact that copper doping leads to significant inhibition of both Gram-positive and Gram-negative bacteria, while maintaining the bioactivity of calcium magnesium silicates.

Samples obtained by combustion demonstrated slightly stronger antibacterial behavior than those synthesized by sol–gel, likely due to their higher surface reactivity and crystalline definition, which facilitate ion release. The presence of Ca^2+^ ions may further contribute to bacterial inhibition by increasing the local pH and osmolarity, leading to membrane destabilization. In contrast, the more amorphous nature and lower surface area of sol–gel-derived scaffolds likely reduced ion availability, explaining the weaker inhibition zones observed. Overall, the antibacterial performance can be attributed to the synergistic effects of copper ions release, scaffold surface reactivity, and bacterial membrane susceptibility, which collectively determine the observed microbial inhibition behavior.

**Figure 12:**
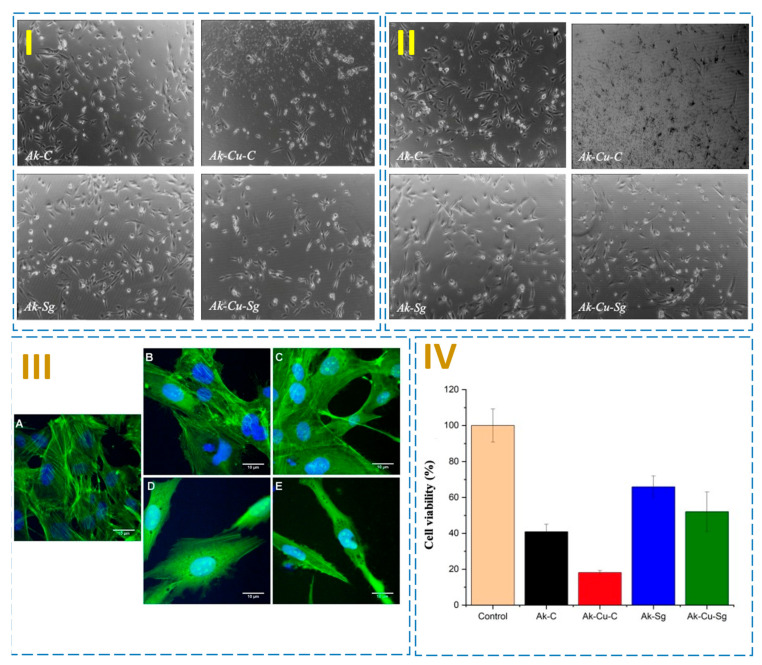
Optical microscopy images after (**I**) 24 h of incubation and (**II**) 48 h of incubation (images taken with an objective with 10× magnification), (**III**) fluorescence microscopy images of cells in contact with (**A**) control (glass), (**B**) Ak-Sg-1200, (**C**) Ak-C-1200, (**D**) Ak-Cu-Sg-1200 and (**E**) Ak-Cu-C-1200 (scale bar is 10 μm for all images) and (**IV**) results of the MTT cell viability test after 24 h of exposure to control (glass), Ak-Sg-1200, Ak-C-1200, Ak-Cu-Sg-1200 and Ak-Cu-C-1200. Data are presented as mean ± standard deviation (*n* = 3). Statistical analysis revealed highly significant differences for all tested samples (*p* < 0.001).

Microscopy images for each sample after 24 and 48 h of incubation are shown in Figure 12I,II. Cells cultured on silicate-based surfaces exhibit a normal morphology, with an elongated shape characteristic to the osteoblast cells. On copper-doped samples, the cells remain adherent to the surface of the material; however, they have a significantly reduced number, especially in the case of the Ak-Cu-C sample after 48 h. These morphological findings are consistent with the viability assay results (Figure 12IV), which showed a clear reduction in cell viability on the copper-doped scaffolds. The lower cell density observed microscopically may therefore be associated with the reduced viability induced by local copper-rich areas resulting from copper segregation, while the remaining cells maintained their adhesion and spreading on the scaffold surface. The work of Kunqiang et al. [63] concluded that the higher the copper concentration, the lower the cell survival rate, and the threshold for the antibacterial efficacy and cytotoxicity should be carefully considered in tissue engineering.

Confocal fluorescence microscopy images (Figure 12III) showed osteoblast-like cells with normal morphology: well-defined nucleus (blue) and organized actin filaments (green) similar to the control sample confirm good biocompatibility of undoped materials. In contrast, copper-doped scaffolds exhibited fewer adherent cells with a more contracted morphology, consistent with moderate cytotoxicity induced by copper release. Nevertheless, viable cells were still observed, indicating that copper incorporation is not fully cytotoxic but requires optimization to balance its antibacterial benefits with cellular compatibility.

The cellular tests aimed to assess the biocompatibility of the undoped and copper-doped scaffolds by evaluating cell viability and adhesion (Figure 12IV). Analysis of the fluorescence images indicates that the morphology of the cells treated with Ak-C-1200, Ak-Sg-1200, and Ak-Cu-Sg-1200 does not differ significantly from that observed under the control condition. No changes at the nuclear level are observed, and the cells appear well spread and properly developed. In contrast, treatment with Ak-Cu-C-1200 results in noticeable morphological alterations, including modifications in nuclear morphology. The MTT viability assays revealed a decrease in cell survival on copper-doped samples compared to undoped ones, most likely due to the cytotoxic effect of elevated copper concentrations [63]. Scaffolds obtained via the sol–gel method supported better cell proliferation and adhesion than those produced by combustion, attributed to their more compact microstructure and slower, more uniform ion release, which reduces the risk of local toxic concentrations. In particular, the Ak-Cu-C-1200 sample exhibited a noticeable reduced viability, indicating a cytotoxic response under the tested conditions. This behavior may be explained by the combined effect of the relatively high copper content and the combustion-derived microstructure, which could promote a burst ion release and thereby increase the effective copper exposure to cells.

Overall, the results highlight that the antibacterial efficacy and cytocompatibility of the scaffolds are strongly interdependent and primarily governed by the copper content, as well as by the phase composition and microstructural features. An optimal balance between these competing effects is therefore essential, since excessive copper incorporation or microstructural conditions that promote rapid ion release may enhance antibacterial activity but simultaneously induce cytotoxic responses. Consequently, careful control of copper concentration, along with phase stability and optimal microstructure is crucial to tailor the biological performance of the scaffolds for tissue engineering applications.

Mechanical properties such as compressive strength and elastic modulus are critical parameters for scaffolds intended for hard tissue engineering applications, as they directly influence load-bearing capability and structural stability. In the present study, no direct mechanical testing was performed; however, the expected mechanical behavior of the investigated materials can be discussed in relation to their compositional and structural characteristics. Compared to dense akermanite ceramics, which are reported to exhibit relatively high compressive strength and stiffness due to their compact microstructure, the present scaffolds are expected to show different mechanical performance. This is attributed to their complex multiphase nature, consisting of binary, ternary, and single oxide/silicate phases, as well as their inherent porosity. Furthermore, the microstructural features are strongly influenced by the processing route (combustion or sol–gel) and the presence of copper doping, which can modify densification behavior, grain connectivity, and overall structural integrity. Therefore, the mechanical response of the current systems is anticipated to differ significantly from that of dense single-phase akermanite, being governed by the combined effects of phase complexity, porosity, and microstructural evolution.

## 4. Conclusions

This study demonstrated the successful fabrication of silicate and copper-doped silicate scaffolds by robocasting for potential use in bone tissue engineering. Akermanite was chosen as a starting point for its high mechanical strength and bioactivity, while copper doping enhanced the antibacterial properties of the scaffolds and may offer angiogenic potential. The 3D printing process enabled precise control of pore size and interconnectivity, and it was established that both the synthesis route and dopant presence affect the paste formulation for the printing process. Comprehensive characterization (TGA, SEM, EDX, FTIR, XRD) confirmed the structural integrity, phase composition, and thermal stability of the scaffolds. The results also indicated the partial incorporation of copper in the lattice of the silicates through substitutional mechanism by both combustion and sol–gel synthesis methods. The sol–gel method produced more homogeneous microstructures and improved bioactivity, whereas combustion-derived samples showed higher crystallinity and ion release. The presence of the copper led to significant inhibition of both Gram-positive and Gram-negative bacteria, while maintaining the bioactivity properties of the silicate scaffolds. Moreover, the obtained materials exhibited favorable cytocompatibility and supported cell attachment and spreading, as evidenced by the MTT assay and microscopy observations.

Overall, integrating metal-doped silicates into additive manufacturing represents a promising strategy for developing multifunctional and bioactive scaffolds. Future research should focus on optimizing synthesis and doping conditions to further enhance the biological performance of silicate-based materials. Additionally, detailed mechanical characterization, including compressive strength and elastic modulus measurements, together with in vivo assessments are essential steps toward validating these scaffolds for clinical trials.

## Figures and Tables

**Table 1:** Identification codes of the investigated samples according to composition, indicating the synthesis method and the nomenclature adopted for the calcined powders and sintered scaffolds.

Sample	Synthesis Method	Code for Calcined Powders	Code for Sintered Scaffolds
Akermanite	Combustion	Ak-C-900	Ak-C-1200
Copper-doped akermanite		Ak-Cu-C-900	Ak-Cu-C-1200
Akermanite	Sol–gel	Ak-Sg-900	Ak-Sg-1200
Copper-doped akermanite		Ak-Cu-Sg-900	Ak-Cu-Sg-1200

**Table 2:** Phase composition of the powders calcined at 900 °C (Ak-C-900, Ak-Cu-C-900, Ak-Sg-900 and Ak-Cu-Sg-900) and scaffolds sintered at 1200 °C (Ak-C-1200, Ak-Cu-C-1200, Ak-Sg-1200 and Ak-Cu-Sg-1200), where C_3_MS_2_—merwinite, C_2_MS_2_—akermanite, CMS_2_—diopside, CMS—monticellite, C_2_S—larnite, MgO—periclase and CuO—tenorite.

Sample	C_3_MS_2_	C_2_MS_2_	CMS_2_	CMS	C_2_S	MgO	CuO
	(wt%)						
Ak-C-900	49.8	27.4	-	-	-	22.8	-
Ak-Cu-C-900	-	-	-	-	65.8	16.4	17.8
Ak-Sg-900	18.8	10.3	27.8	-	34.5	8.6	-
Ak-Cu-Sg-900	23.1	12.7	-	-	42.1	10.6	11.5
Ak-C-1200	89.4	-	-	4.9	-	5.7	-
Ak-Cu-C-1200	-	-	-	-	57.1	26.8	16.1
Ak-Sg-1200	50.2	40.0	9.8	-	-	-	-
Ak-Cu-Sg-1200	68.6	7.3	-	15.6	-	-	8.5

**Table 3:** Diameter of the inhibition zone of the scaffolds sintered at 1200 °C (Ak-C-1200, Ak-Cu-C-1200, Ak-Sg-1200 and Ak-Cu-Sg-1200) against *E. coli*. and *B. subtilis* after 24 h of incub.

Sample	m (g)	IZ (mm)	IZ_eff * (mm)
***Escherichia coli***			
Ak-C-1200	0.120	2.00	1.92
Ak-Cu-C-1200	0.145	6.00	6.95
Ak-Sg-1200	0.120	0.00	0.00
Ak-Cu-Sg-1200	0.116	6.00	5.56
***Bacillus subtilis* spizizenii Nakamura**			
Ak-C-1200	0.162	0.00	0.00
Ak-Cu-C-1200	0.135	3.00	3.27
Ak-Sg-1200	0.130	0.00	0.00
Ak-Cu-Sg-1200	0.123	0,67	0.60

* IZ_eff is IZ reported to the tested amount of sample.

## Data Availability

The original contributions presented in the study are included in the article; further inquiries can be directed to the corresponding author.
